# Genome-wide analysis of the distribution of AP2/ERF transcription factors reveals duplication and *CBFs* genes elucidate their potential function in *Brassica oleracea*

**DOI:** 10.1186/1471-2164-15-422

**Published:** 2014-06-03

**Authors:** Senthil Kumar Thamilarasan, Jong-In Park, Hee-Jeong Jung, Ill-Sup Nou

**Affiliations:** 1 Department of Horticulture, Sunchon National University, 413 Jungangno, Suncheon, Jeonnam 540-950, Republic of Korea

**Keywords:** *Brassica oleracea* AP2/ERF, Classification, Gene expression, Abiotic stresses, *BoCBF* genes

## Abstract

**Background:**

Cabbage (*Brassica oleracea*) is one of the most important leaf vegetables grown worldwide. The entire cabbage genome sequence and more than fifty thousand proteins have been obtained to date. However a high degree of sequence similarity and conserved genome structure remain between cabbage and *Arabidopsis*; therefore, *Arabidopsis* is a viable reference species for comparative genomics studies. Transcription factors (TFs) are important regulators involved in plant development and physiological processes and the AP2/ERF protein family contains transcriptional factors that play a crucial role in plant growth and development, as well as response to biotic and abiotic stress conditions in plants. However, no detailed expression profile of AP2/ERF-like genes is available for *B. oleracea*.

**Results:**

In the present study, 226 AP2/ERF TFs were identified from *B. oleracea* based on the available genome sequence. Based on sequence similarity, the AP2/ERF superfamily was classified into five groups (DREB, ERF, AP2, RAV and Soloist) and 15 subgroups. The identification, classification, phylogenetic construction, conserved motifs, chromosome distribution, functional annotation, expression patterns and interaction network were then predicted and analyzed. AP2/ERF transcription factor expression levels exhibited differences in response to varying abiotic stresses based on expressed sequence tags (ESTs). *BoCBF1a, 1b, 2, 3* and *4*, which were highly conserved in *Arabidopsis* and *B. rapa CBF*/*DREB* genes families were well characterized. Expression analysis enabled elucidation of the molecular and genetic level expression patterns of cold tolerance (CT) and susceptible lines (CS) of cabbage and indicated that all *BoCBF* genes responded to abiotic stresses.

**Conclusions:**

Comprehensive analysis of the physiological functions and biological roles of AP2/ERF superfamily genes and *BoCBF* family genes in *B. oleracea* is required to fully elucidate AP2/ERF, which will provide rich resources and opportunities to understand abiotic stress tolerance in crops.

## Background

Plant growth, development and yield are influenced greatly by numerous biotic and abiotic stresses, including cold, salt, drought and soil salinization. Plant stresses are generally controlled by a network of specialized genes through intricate regulation by specific transcription factors (TFs). The AP2/ERF superfamily, which is one of the largest groups of TFs families, is involved in the regulation of plant developmental processes and biotic and abiotic stress responses [1,2]. This family includes all genes encoding at least one APETALA2 (AP2) domain and can be further separated into ETHYLENE RESPONSE FACTOR (ERF), AP2, RAV and Soloist families. The ERF family encodes proteins with a single AP2 domain, while the AP2 gene family codes for TFs with two AP2 domains [3,4]. Lastly, the RAV family encodes proteins processing a single AP2 domain plus an additional B3 domain, which is also present in other, non ERF- transcription factors [5].

To date, two major efforts to define a nomenclature for ERF sequences have been published. Sakuma *et al*. [6] divided the *Arabidopsis* ERF family into two subfamilies based on the amino acid sequence of the DNA binding domain, the *CBF/DREB* subfamily (group A) and ERF subfamily (group B). These two families were then further subdivided into six groups [7]. Conversely, Nakano *et al*. [4] divided the AP2/ERF domain amino acid sequences, of *Arabidopsis* and rice ERF families into 12 and 15 groups, respectively. Similarly, 10 groups were identified in the grape and cucumber ERF family [3].

After release of the whole genomic sequences of several plant organisms, a variety of AP2/ERF TFs were successfully identified and investigated in several plants, including *Arabidopsis*, rice [4,8], grape [3], poplar (*Populus tricocarpa*) [9], wheat (*Triticum aestivum*) [10], cucumbers [5], barley (*Hordeum vulgare*) [11], and soybean (*Glycine max*) [12]. The AP2/ERF TFs regulate diverse biological processes in plant function and development, such as hormones, reproduction, cell proliferation, and abiotic and biotic stress responses [13,14].

The DREB subfamily is commonly used as a viable candidate to enhance crop abiotic stress tolerance. The DREB subfamily exhibits different response patterns under environmental stress, including low-temperature (*AtCBF1*) [15], drought (*OsDREB1*) [16,17], and high salinity (*CaDREBLP1*) [18]. The DREB TFs activate multiple dehydration/cold-regulated genes by interacting with DRE/CRT elements (A/GCCGAC), which are present in the Cold response/Responsive to desiccation COR/RD gene promoters [19]. In addition, several DREB subfamily genes are reportedly positive and negative mediators of ABA and sugar responses, primarily during the germination and early seedling stages [20]. Therefore, the *CBF*/*DREB* family genes were annotated and a qRT-PCR platform that allows investigation of their expression profile was developed in the present study.

Cabbage (*Brassica oleracea*) is one of the most popular vegetable crops worldwide. Currently, *Brassica* crops are used for human consumption and provide resources for investigation of genome evolution of cabbage [21]. The *B. oleracea capitata* (line 02–12) genome was recently sequenced and assembled, http://www.ocri-genomics.org/bolbase/ [22]. Release of the entire cabbage genome sequence, as well as those of *Arabidopsis* and *B. rapa* have provided an opportunity for comparative genomic study of AP2/ERF TFs. Characterization of AP2/ERF superfamily genes in *B. oleracea* can clarify the molecular mechanisms responsible for abiotic stress responses to conditions such as cold, salt, drought and ABA, which will enable development of, *Brassica* varieties with increased tolerance to many adverse environments using transgenic technology. In the present study, 226 putative TFs in the AP2/ERF family were identified from the database of the *B. oleracea* genome. Bioinformatic methods were used to analyze the sequence information and construct a phylogenetic tree. Some gene duplication events of AP2/ERF family TFs in *B. oleracea* were found. Identified *B. oleracea CBF*/*DREB* genes were then selected for quantitative real-time PCR analysis, to determine the expression level of TFs under different abiotic stresses.

## Results and discussion

### Identification of the AP2/ERF family TFs in *B. oleracea*

The availability of complete *B. oleracea* genome sequences has made it possible to identify all of the AP2/ERF family members in cabbage for the first time. To identify the four family genes, we conducted extensive BLASTN searches based on *B. rapa* and *A. thaliana* nomenclature suggestions [23,7]. Our extensive search for AP2-domain containing proteins identified 226 distinct AP2/ERF putative TFs (Additional file 1: Table S1). A total of 181 genes with a single AP2/ERF domain were assigned to the ERF family. Additionally, 32 genes were grouped into the AP2 family, based on the presence of a tandem repeated double AP2/ERF motif. The RAV family included 13 genes identified as encoding one AP2/ERF domain together with one B3 domain. The remaining genes, Bol184 and Bol185 were not only divergent from the ERF family, but were homologous with *Arabidopsis* and *B. rapa* Soloist (*Br265* and *At127*, respectively) and therefore designated as Soloist.

Previous annotations of AP2/ERF genes nomenclature proposed by Sakuma *et al.*[6] were based on homology of the DNA binding domain alone. However, Nakano *et al.*[4] proposed an alternative method, based on the presence of domains that were different from the DNA binding domain. Therefore, we subdivided the cabbage AP2/ERF genes into 15 groups based on conserved domain similarities to *B. rapa* and *Arabidopsis* AP2/ERF TFs. Cumulatively, the number of AP2/ERF TFs in cabbage exceeded that in *Arabidopsis* (147) poplar (202), and rice (196) [7]. The proportion of each subfamily is shown in Table 1 and Additional file 2: Figure S1. The RAV family number in cabbage (13) was larger than that in other plant groups, including *Arabidopsis* (6), rice (4), and tomato (3), but lesser than that of *B. rapa* (14). Cabbage contained more AP2 double domain genes in each subgroup than *Arabidopsis*, and the DREB A-4 subgroup had up to two times more than *Arabidopsis*.

**Table 1:** **Summary of AP2/ERF transcription factors of cabbage, *Arabidopsis *** **and Chinese cabbage**

**Plant**		***Brassica oleracea *** **Number**	***Arabidopsis thaliana *** **Number**	***B. rapa *** **subsp. *Pekinensis *** **Number**
**Classification**	**Group**			
**DREB subfamily**	A1	8	6	10
	A2	9	8	20
	A3	1	1	5
	A4	33	16	35
	A5	23	16	27
	A6	17	10	15
	**Total**	**91**	**57**	**112**
**ERF subfamily**	B1	19	15	22
	B2	10	5	10
	B3	25	18	35
	B4	5	7	14
	B5	19	8	28
	B6	10	12	23
	**Total**	**88**	**65**	**132**
**AP2 subfamily**		32	18	30
**RAV subfamily**		13	6	14
**Soloist**		2	1	1
**Total AP2/ERF family factors**		**226**	**147**	**289**

### Phylogenetic analysis of AP2/ERF TFs family

**Figure 1:**
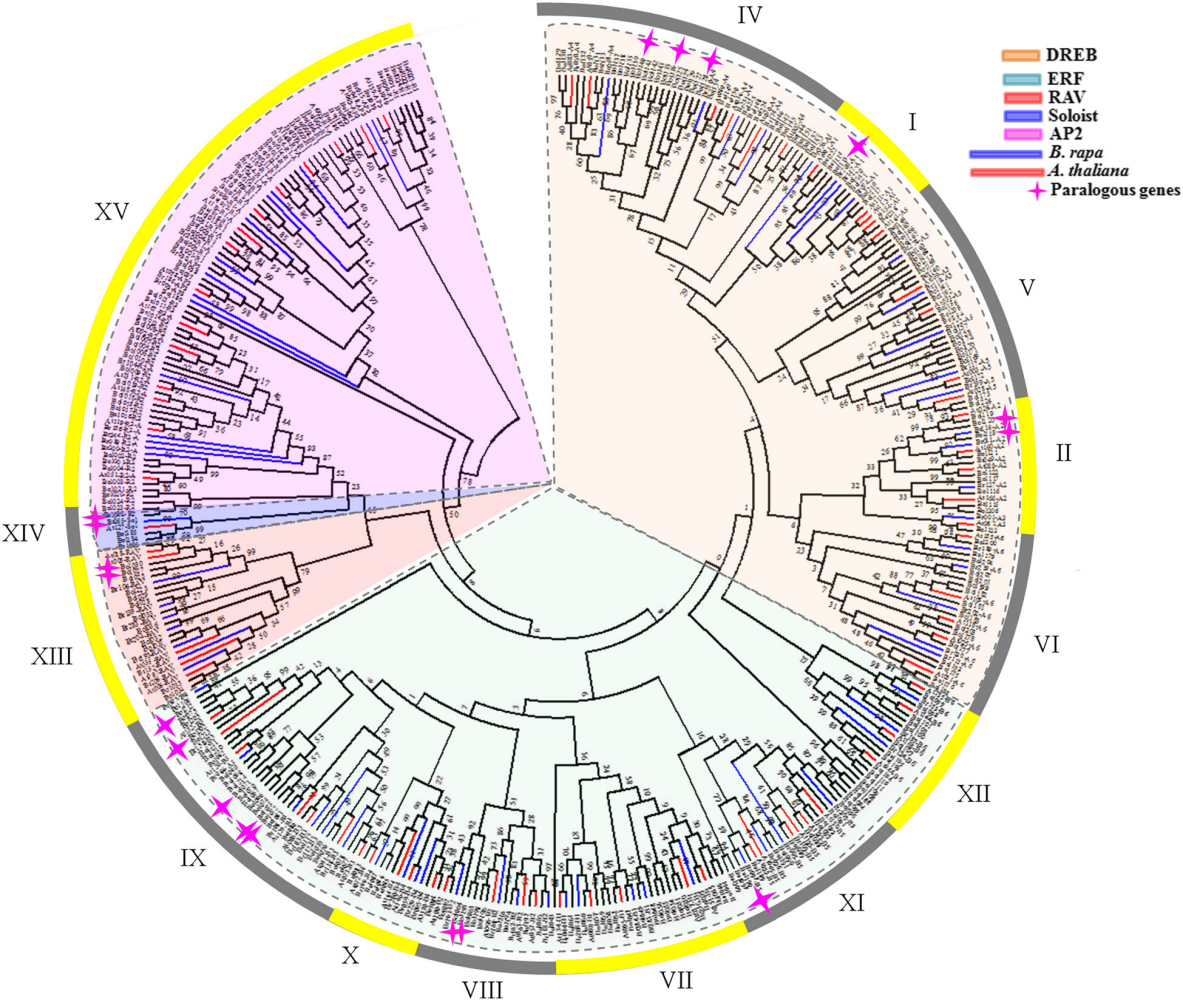
**Phylogenetic tree constructed by the neighbor-joining method using AP2/ERF transcription factor domains in cabbage, Chinese cabbage and *Arabidopsis*.** The tree was divided into 15 groups that contained ERF (DREB and ERF subfamily), AP2, RAV and the Soloist family. The star represents paralogous genes of cabbage. Lines represent Chinese cabbage (blue) and *Arabidposis* (red).

To investigate the evolutionary relatedness of the identified sequences together with AP2/ERF genes encoded by the other fully sequenced Chinese cabbage and *Arabidopsis*, we performed phylogenetic reconstruction using the conserved AP2/ERF transcription factor domain. The resulting phylogenetic tree (Figure 1) resolved 15 clades containing the ERF, AP2, RAV and Soloist families, which was in accordance with previous studies [7,23]. Groups I to VI represent the DREB subfamily, while groups VII to XII represent the ERF subfamily and groups XIII, XIV and XV indicate the AP2, RAV and Soloist families, respectively. Although the Soloist transcription factor contained a single AP2 domain in cabbage, it clustered with the RAV family, while the Soloist transcription factor grouped with the AP2 family in grape [3]. We conducted a more in depth phylogenetic analysis of the AP2 family by selecting the AP2 family proteins, which contained two AP2 domains. Chinese cabbage and *Arabidopsis* were divided into two groups, AP2-R1 and AP2-R2, respectively (Additional file 2: Figure S2).

The conservative motifs among AP2/ERF proteins in plants were clarified by conducting multiple alignment analyses using amino acid sequences of the AP2/ERF domain. Several genes were selected from each species of the AP2/ERF family to identify the motifs. Sequence alignment showed that the motif length in the RAV and Soloist family was longest, and contained 50 amino acids, followed by DREB (41 amino acids). However, the AP2 family contained two groups (AP2-R1 and AP2-R2), that might be responsible for the reduced motif length (29 amino acids). The divergence between the two groups might affect the AP2 family motif length, with a higher divergence generally being associated with a shorter motif. The same explanation might be used for the ERF subfamily (26 amino acids), which contained six groups, and a shorter conserved motif. Although six groups were identified in the DREB and ERF subfamilies, the conserved motif was much longer than in the ERF subfamily (Additional file 2: Figure S3). The differences within the AP2/ERF family were further analyzed by examining the DREB, ERF and AP2 conserved motifs using the MEME program. The results showed that all but two (DREB-A2 and DREB-A6) of the six groups had a 50 amino acid conserved motif, which contained 32 and 29 amino acids in the conserved motif, respectively (Additional file 2: Figure S4). In the ERF subfamily, a shorter conserved motif was observed in ERF-B2, B3, B5 and B6 than in the remaining ERF group (50 amino acids). Therefore, if the ERF subfamily motif length was dependent on the ERF-B5 group, it might be responsible for the overall shorter ERF subfamily motif. The AP2-R2 group contained 29 amino acids identified in the AP2-R1 group conserved motif (Additional file 2: Figure S5 and Figure S6).

Sequence alignment of all AP2/ERF families indicated that LG, AA and YD elements were highly conserved (Additional file 2: Figure S3). The WLG element in DREB, ERF and RAV was more highly conserved than that in AP2 and Soloist. In the Soloist family, some WLG elements were converted into HLG elements. The AYD element was conserved in the AP2/ERF superfamily, with the exception of Soloist, where it was converted into LYD (Additional file 2: Figure S3). We assume that specific conserved motifs are related to molecular functions. We use this assumption as an effective and practical means to predict unknown protein functions were derived from structural relationship in *Arabidopsis*[4].

### Chromosome distribution of the AP2/ERF TFs family

**Figure 2:**
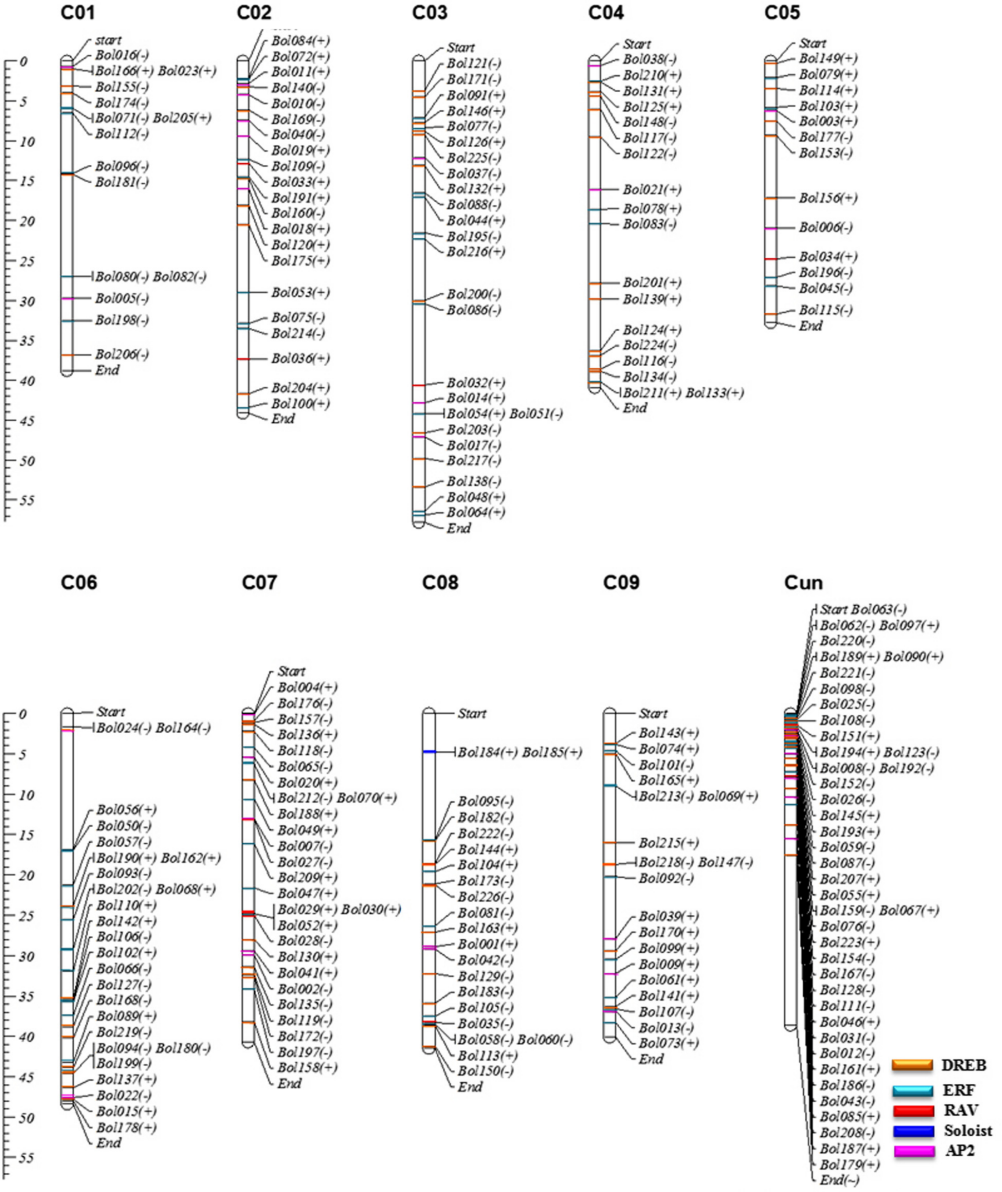
**Distribution of 226 AP2/ERF genes on the nine cabbage chromosomes.** Forty one genes on the Cun (Scaffold) could not be anchored onto a specific chromosome. Scales are in megabases (Mb).

Among all AP2/ERF family TFs resolved in the cabbage genome, 91 genes belong to the DREB subfamily, followed by 88 in ERF, 32 in AP2, 13 in RAV and two belonging to Soloist (Table 1 and Additional file 2: Figure S1). A total of 185 AP2/ERF TFs were distributed on nine cabbage chromosomes (Figure 2 and Additional file 1: Table S3), and while 41 genes could not be assigned to any specific chromosome. Chromosome 6 and 7 had the highest number of AP2/ERF TFs (26 and 27, respectively), while the lowest AP2/ERF transcription factor number was found on chromosome 5 (13 genes) and 1 (15 genes). The high AP2/ERF sequence number on chromosomes 6 and 7 was primarily due to the increased number of DREB (10 and 9) and ERF (13 and 9) subfamilies. Interestingly, conserved sequences and the physical proximity of repetitive TFs, that belonged to the same group were identified and located on the same chromosomal regions as follows: *Bol010* to *Bol011* and *Bol018* to *Bol019* (XV group) were located on chromosome 2; *Bol116* to *Bol117* (II group), *Bol124* to *Bol125* (IV group) and *Bol210* to *Bol211* (IX group) were located on chromosome 4; *Bol118* to *Bol119* (II group), *Bol135* to *Bol136* (IV group), *Bol157* to *Bol158* (V group) and *Bol027 to Bol030* (XIII group) were located on chromosome 7; *Bol056* to *Bol057* (VII group) and *Bol093* to *Bol094* (XI) were located on chromosome 6; *Bol182* to *185* (VI, XIV) and *Bol104* to *Bol105* (XII) were located on chromosome 8; *Bol073* to *Bol74* (IX group) were located on chromosome 9 (highlighted in Additional file 1: Table S1). Similar patterns were also found in the *Arabidopsis*[6], *B. rapa*[23], grape and poplar genomes [3,10], which were suggested to represent paralogous segments resulting from ancestral polyploidization events. The highest number of RAV TFs was found on chromosome 6 (4 genes), followed by chromosomes 2, 3, 5, 8 and Cun (2, 1, 1, 1 and 4 genes respectively), while RAV TFs were not detected on chromosomes 1, 4, 7 and 9. Moreover, 17.5 and 19.3% of the DREB and ERF TFs were observed on chromosome scaffolds (Additional file 1: Table S3).

We also investigated the cabbage paralogous AP2/ERF TFs. A total of 198 AP2/ERF TFs showed high homology (>85%) to the AP2/ERF genes (Additional file 1: Table S4). The paralogous genes were indicated in the phylogenetic tree (Figure 1). Duplication events have been studied in grape, and 17 proteins with high similarity sequences were reported [3]. In the present study, we identified 14 genes in the cabbage genome with 100% sequence similarity. Among the 14 duplicated genes, eight were ERF-subfamily members, while the rest belong to the DREB subfamily (4 genes) and RAV subfamily (2 genes) (Additional file 1: Table S4).

### AP2/ERF TFs expression patterns in *B. oleracea*

**Figure 3:**
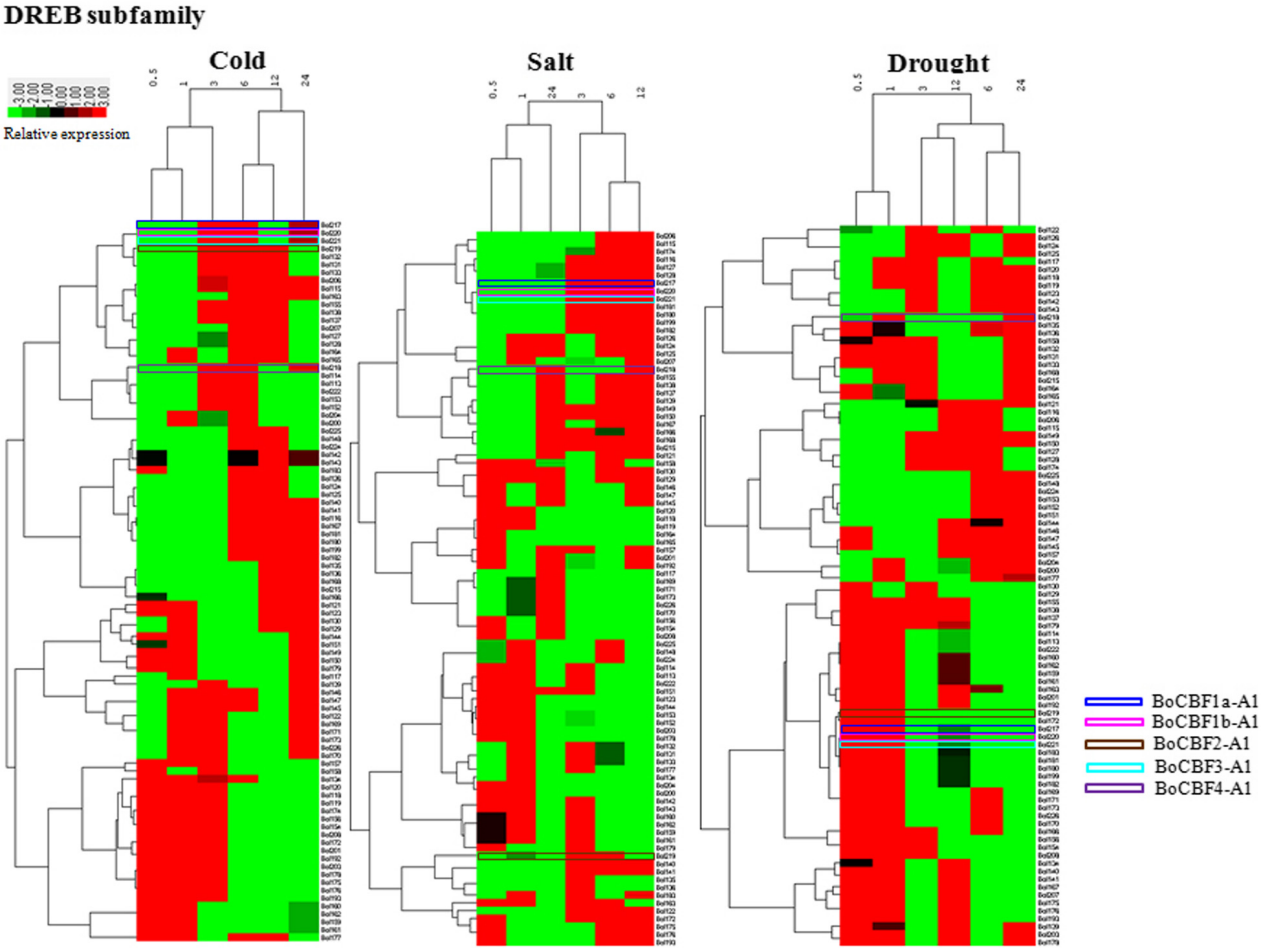
**Expression profile cluster analysis of the cabbage ERF subfamily proteins.** The expression values of each ERF subfamily gene identified in the study were measured by EST tags from cold, salt and drought.

**Figure 4:**
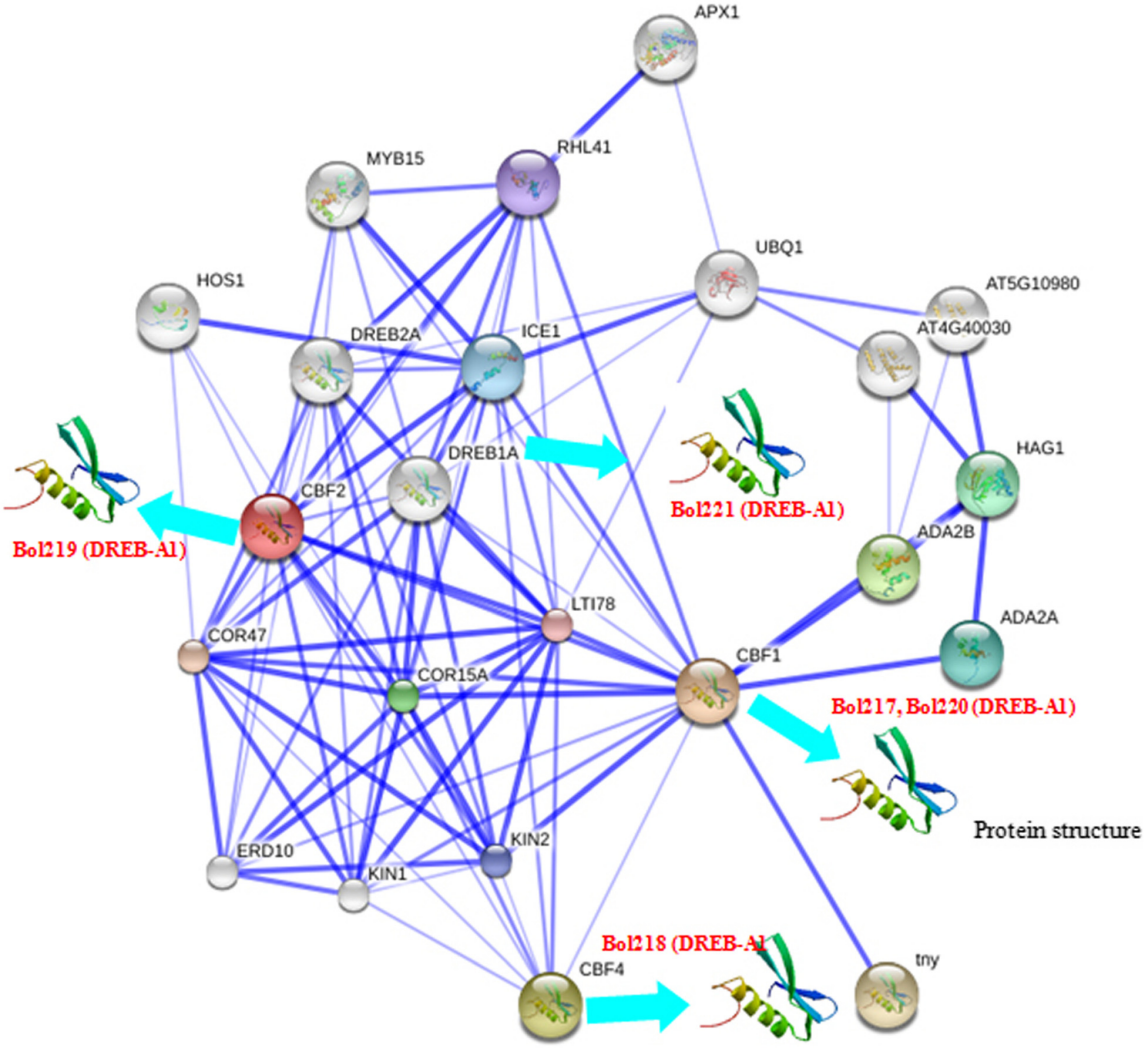
**Interaction network of five BoCBF genes identified in cabbage and related genes in *Arabidopsis.*** Stronger associations are represented by thicker lines.

We also investigated the expression patterns of *B. oleracea* AP2/ERF genes during abiotic stress condition, such as cold, salt and drought using the microarray data. In this study, based on *Arabidopsis* BLAST hits, cold, salt and drought expression profiles were downloaded from the AtGenExpress visualization tool to evaluate responses to stress [24]. The results provided rich resource for gene discovery and investigation of gene expression patterns. A total of 221 AP2/ERF TFs were obtained by expression profile tags (Additional file 1: Table S5). Most AP2/ERF proteins belonged to the DREB and ERF subfamily. In the DREB family, gene relative expression profiles exhibited in all time courses had a high degree of expression pattern such as up (red) and down (green) - regulated genes. A few genes were not expressed (black) in response to any stress or throughout the study period (Figure 3). Detailed expression values and clusters of each AP2/ERF family transcription factor were analyzed using cluster analysis based on *Arabidopsis* best hits of gene expression value (Additional file 2: Figure S7- S9). Conversely, the predicted *B. oleracea* AP2/ERF genes were annotated based on sequence alignment to non-redundant (nr) public databases using BLASTN. Each AP2/ERF was annotated from the nr database was integrated and results provided in a supplemental file (Additional file 1: Table S6). Interestingly, we identified five CBF proteins with the highest similarity to *Arabidopsis* protein might be related to cold and freezing tolerance in *B. oleracea* from Bolbase database using BLASTN. Cellular localization is often an important factor in determining protein function. Liu et al. [25] has suggested that TFs are located only in the nucleus. PSORT was applied to predict and determine localization of BoCBF proteins was in the nucleus. Gene structure revealed that the all *BoCBFs* had almost no introns. It was one of the representative characteristics of the CBFs family members, CBF proteins were well characterized listed in the Table 2, Additional file 2: Figure S10 [26], and all five of the aforementioned genes were identified as the members of the DREB-A1 group. Subsequently, cabbage and *Arabidopsis* protein interactions, including functional and physical interactions were examined using the STRING software and the corresponding database to identify the protein interactions [27]. Five proteins that exhibited increased sequence similarity to CBF1 (Bol217, Bol220) and CBF2 (Bol219) were involved in stronger (thicker lines) interaction network. The Bol218 protein, which showed high homology with CBF4, was involved in weak interactions (thinner lines) with all genes (Figure 4). The former network largely participated in cold regulatory pathways, as most factors were related to cold stress, including COR47, COR15A, KIN2, CBF1, CBF2, and ADA2A [28]. Many homologues are known to be induced rapidly upon exposure to low temperature, such as the *AtCBF1-3* genes from *Arabidopsis*[29], and the *ZmDREB1A* gene from *Zea mays*[30]. *At*CBF4 is not induced by cold, but the *CBF4* network might be involved in drought adaptation [31,32].

**Table 2:** **Amino acid sequence characteristics of *BoCBF *** **genes**

**Gene ID**	**Sequenced ID ( *Bol *** **base)**	**TAIR homology (aa) (%Id)**	**Nt (bp) length**	**Chr. no., position**	**aa length**	**pI**	**Mol. wt(kDa)**	**α-helix (%)**	**β- sheet (%)**	**NLS**		**Subcellular localization**
										**Monopartite**	**Bipartite**	
*BoCBF1a*	Bol029967	AT4G25490 (68)	645	C03; 49752542–49753186 (-)	214	5.03	23.77	32.71	7.94	65-79	-	n/13.0
*BoCBF1b*	Bol006459	AT4G25490 (59)	753	Cun; 000278: 15959–16711 (-)	250	4.85	27.86	30.80	11.20	64-78	33-60, 196-228	n/12.0
*BoCBF2*	Bol042239	AT4G25470 (62)	648	C07; 43885424–43886071 (-)	215	5.34	23.82	30.23	10.23	66-80	-	n/11.0
*BoCBF3*	Bol006460	AT4G25480 (50)	834	Cun; 000278: 49871-50704	277	4.58	30.45	36.10	9.75	68-82	224-255	n/10.0
*BoCBF4*	Bol029422	AT5G51990 (68)	663	C09; 18733153-18732491	220	6.14	24.51	35.45	9.09	40-49, 69-83	-	n/12.0

aa- amino acid; NLS- nuclear localization signal; n- nucleus.

**Figure 5:**
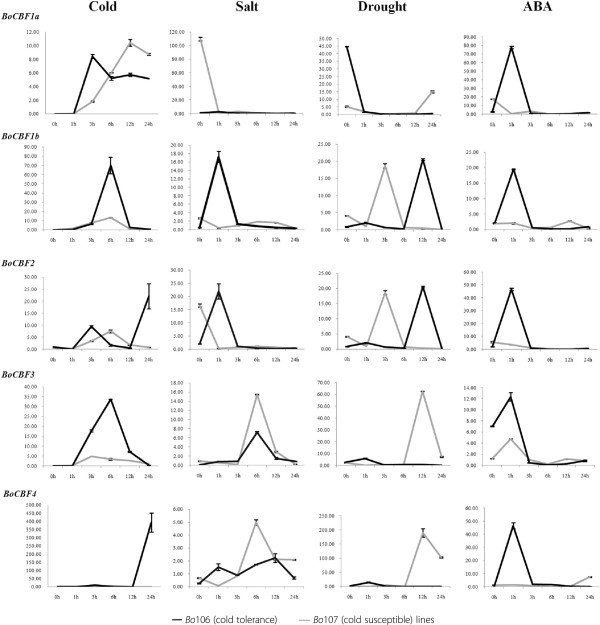
**Relative quantitative (RQ) expression levels of five *BoCBF *genes at a series of time points following the abiotic stress treatments.** Range between minimal and maximal Ct value representing expression levels of each candidate gene were analyzed using qbasePLUS. ─ *Bo*106 (cold tolerance) and *Bo*107 (cold susceptible) lines.

There were a large number of DREB- and ERF-type sequences related to stress. We obtained five *BoCBF* family genes based on the *Arabidopsis* protein interaction. Quantitative real-time PCR was used to analyze the expression profile of *CBF*/*DREB* family genes under cold, salt, drought and ABA stresses using two contrasting genotype lines: cold tolerant *Bo*106 (CT) and cold susceptible *Bo*107 (CS). All genes analyzed exhibited differential accumulation in response to cold, salt, drought and ABA (Figure 5, Additional file 3).

Evaluation of *CBF* gene expression revealed that some *CBF* genes in plant species could be induced by short term exposure to cold stress [33]. In our study, five novel *BoCBF* genes showed different expression patterns when the plants were exposed to cold stress. Detected transcripts, *BoCBF2* and *BoCBF4* were negatively regulated by cold stress; however, the patterns among them were not the same. Specifically, *BoCBF4* was more highly up-regulated at 24 h than *BoCBF2*, while *BoCBF2* was elevated at 3 h, then declined after 6 h, but was maintained at a level higher at 24 h. *BoCBF1b* and *BoCBF3* were also negatively regulated by cold stress. *BoCBF1b* was more highly elevated at 6 h than *BoCBF3*. However, *BoCBF1b* and *BoCBF3* had a similar expression pattern after 6 h, which might be resistant to cold stress. *BoCBF1a* was regulated by both positive and negative at 3 h. CT line was more highly regulated at 3 h than CS, but was maintained at a level higher at 12 h. Overall, these findings indicate that *BoCBF* genes might be involved in mediating cold signaling transduction when plants are exposed to cold temperature. However, Haake *et al.*[33] and Medina *et al*. [26] reported that *CBF* gene expression was modulated by salt and dehydration. *BoCBF3* and *BoCBF4* were positively regulated by salt stress; however, they showed similar expression patterns and maintained the high abundance at 6 h. *BoCBF1b* and *BoCBF2* had similar expression pattern were negatively regulated by salt stress, which are shortly induced at 1 h followed by gradual decrease after 3 h which might be responsible resistant to salt stress. It should be noted that *BoCBF* genes showed stress responsive (positive or negative) at 1 h, indicating that they play an important role in mediating salt signal transduction when plants are exposed to salt stress at short time. *BoCBF1b* and *BoCBF2* had differential expression pattern, however, they had similar expression. CS and CT line has maintained the high abundance at 3 h and 12 h in both the genes respectively. *BoCBF1a, BoCBF3* and *BoCBF4* were positively regulated by drought stress. However, these genes were not the same responses as at cold and salt stress. *BoCBF3* and *BoCBF4* were gradually elevated at 12 h, after which it declined. These results indicate that *BoCBF* genes were dramatically influenced by drought stress, indicating, that they are involved in drought signal transduction through a positive regulation pathway. Hong *et al.*[34] and Kirch *et al.*[35] suggested that most cold-regulated genes are also responsive to ABA. In contrast, Medina *et al.*[26] reported, that *CBF* transcripts did not accumulate in response to ABA. Hence, it is necessary to determine whether the accumulation of CBF genes was specifically regulated by cold, salt and drought or was also influenced by ABA stress. Here, all *BoCBF* genes were shortly induced and negatively regulated by ABA stress. *BoCBF3* was showed similar expression pattern between CT and CS lines, which may not be response to ABA stress. *BoCBF3* also showed a similar expression pattern between the CT and CS lines, although ABA resistance gradually decreased than relative to the CT line. These findings indicate that *BoCBF1a*, *BoCBF1b*, *BoCBF2* and *BoCBF4* transcripts were negatively regulated in the ABA signaling pathway. On the other hand, Andersen *et al*. [36] was suggested that, estimate the overall expression variation of the candidate genes and variation between the samples subgroups of the sample set. The candidates with lowest intergroup and intragroup variations give the lowest stability (*S*) value and are therefore ranked higher as more stable. We identified as the most stable expression in cold *BoCBF3*_S (0.010), salt BoCBF1b_S (0.029), drought BoCBF2_S (0.043) and ABA BoCBF3_S (0.010) (detailed see in Additional file 3). This data indicates significantly more reliable qRT-PCR analysis. Therefore, some of the novel *BoCBF* genes identified in our study could possibly be used as target genes to improve plant resistance to abiotic stresses such as cold, salt, drought and ABA. It will be useful to evaluate the functions of the *BoCBF* genes, to generate transgenic crops with tolerance to abiotic stresses in the future.

## Conclusions

In summary, we identified 226 AP2/ERF TFs in the cabbage genome and characterized their expression patterns in response to different abiotic stresses. To date, this is the first comprehensive and systematic investigation of cabbage AP2/ERF TFs. We conducted quantitative RT-PCR analysis of some of the novel *B. oleracea CBF* genes identified in this study and found that they have the potential for use as target genes to improve plant resistance to abiotic stresses, such as cold, drought, salt and ABA in two contrasting genotypes of cabbage. It will be useful to evaluate the functions of the *BoCBF* genes to generate transgenic crops tolerant of abiotic stresses. Overall, our genomic and bioinformatic analysis of the five family TFs and proteins presented in this work will provide an important foundation for further functional evaluations under different stress conditions.

## Methods

### AP2/ERF superfamily transcription factor identification

A search of the *B. oleracea* genome database [22] was conducted to identify all members of the AP2/ERF family. A double strategy to obtain every gene of the AP2/ERF family in the genome was used. The genome DNA database in *B. rapa* subsp. *Pekinensis* was downloaded from the *Brassica* database website (http://brassicadb.org/brad/) [37,38]. The sequences of all AP2/ERF superfamily members in the genome of other species assessed were downloaded from the plant TFDB database (http://planttfdb.cbi.edu.cn/) [39,40] and the amino acid sequence of one or most representative members (i.e. the maximum number of different conserved motifs distinctive of each group) for each group defined by Nakano *et al.*[4] were used as queries to search the Bolbase database. Every sequence identified was subsequently checked against the *Arabidopsis* and *B. rapa* databases to confirm that it belonged to the AP2/ERF superfamily. As a final quality check, we confirmed the presence of the AP2 domain in every AP2/ERF *B. oleracea* gene candidate using SMART [41]. Each subfamily motif was identified using the MEME program [42]. The physical distribution of AP2/ERF genes on chromosomes was drawn by MapChart based on gene position in the genome [43].

### Phylogenetic tree construction

Phylogenetic and molecular evolutionary analyses were conducted using MEGA5.1 [44]. The retrieved conserved domains of AP2/ERF proteins were used to construct phylogenetic trees. The neighbor-joining method was applied to construct different AP2/ERF transcription factor domain trees, using the pair-wise deletion option. Tree reliability was assessed using 1000 bootstrap replicates and the numbers indicated for each clade represent bootstrap support values given as percentages. The data matrix and resulting trees were deposited in TreeBASE (accession S15847, http://purl.org/phylo/treebase/phylows/study/TB2:S15847?x-access-code=171776387c9f3d2134e04184bd5277dc&format=html).

### AP2/ERF superfamily transcription factor expression patterns in *B. oleracea*

*B. oleracea* AP2/ERF superfamily CDS (coding domain sequence) was used to search against the TAIR database using the BLAST tool. The eligible hits (E-value <1e-5, Identity >90%) were selected for each *B. oleracea* AP2/ERF superfamily transcription factor. Based on *Arabidopsis* eligible hits, cold, salt and drought expression data were downloaded from the AtGenExpress visualization tool (AVT) (http://jsp.weigelworld.org/expviz/expviz.jsp). The AP2/ERF protein expression cluster from each stress was analyzed via the Cluster program (http://bonsai.hgc.jp/~mdehoon/software/cluster/software.htm), and results were shown using the Tree View software (http://jtreeview.sourceforge.net/).

### Identification of AP2/ERF annotation and specific gene interaction networks

Cabbage AP2/ERF genes were used as a query to search against available public databases (e-value 1.0E-3) using Blast2go [45], which is a sequence-based tool to assign GO terms and annotation for each BLAST hit obtained by mapping the extant annotation associations [46]. The GO terms for each of the three main categories (biological process, molecular function and cellular component) were obtained from sequence similarity using the default parameters. From these annotations, the 2^nd^ Group level and multilevel GO terms were based on the biological process, molecular function and cellular components. This annotation was simplified and focused on plant related functional categories using Plant GOslim. Specific protein interactions were constructed by applying the STRING software (Search Tool for the Retrieval of Interacting Genes/Proteins, http://string-db.org/) [27].

### Plant materials, abiotic stress conditions

For the expression study, *Bo*106 and *Bo*107 cold tolerance (CT) and susceptible lines (CS) of cabbage seeds were grown aseptically on half-strength MS agar medium in a culture room under a 16 h light photoperiod at 25°C. After three weeks of growth, the seedlings were transferred to fresh liquid MSH (half-strength MS medium without sucrose) medium containing 250 mM NaCl and 100 mM abscisic acid (ABA) for 24 h. To induce cold stress, the seedlings were maintained at 4°C for 24 h. Drought treatment was then applied by keeping the seedlings on the filter paper at 28°C for 24. The samples were then subjected to all stresses for 0 (wild type), 1, 3, 6, 12 and 24 h. Next, the samples were frozen immediately in liquid nitrogen and stored at -80°C until RNA isolation. The total RNA was extracted from the frozen samples of the roots, stems, leaves and flower buds of healthy plants and those exposed to abiotic stress using an RNeasy mini kit (Qiagen, USA), after which RNA was treated with RNase-free DNase (Promega, USA) to remove genomic DNA contamination.

### RT- PCR and real time PCR analyses

RT-PCR was performed using an Avian Myeloblastosis Virus (AMV) one step RT-PCR kit (Takara, Japan). The specific primers for the *BoCBF* genes are listed in the Additional file 1: Table S7. RT-PCR was performed using 50 ng cDNA of plants exposed to various abiotic stresses, including cold, salt drought and ABA for 0, 1, 3, 6, 12 and 24 h. Next, PCR samples with a total volume of 20 μl containing 1 μl of first-strand cDNAs and 2 μl of primers were subjected to 30 cycles of 30 s denaturing at 94°C, annealing at 58°C for 30 s and extension at 72°C for 45 s. The PCR products were electrophoresed on a 2% agarose gel, after which real-time PCR was performed on an Illumina Eco real time machine (PhileKorea Technology, Korea) using 1 μl of first-strand cDNA mixed with SYBR Premix Ex TaqTM (Toyobo, Japan). The thermal cycling conditions were 30 s at 95°C, followed by 40 cycles of 5 s at 95°C and 31 s at 58°C. All Ct values were collectively analyzed by qbase PLUS version: 2.6.1, Biogazelle, Belgium) and by NormFinder (version 0.953, http://www.multid.se/genex/hs410.htm) software following published procedures [47,36]; qbasePLUS manual, Biogazelle, Belgium] to compute gene expression stability values (NormFinder).

### Availability of supporting data

The AP2/ERF transcription factors of *B. oleracea, B. rapa* and *A. thaliana* set supporting the results of this article is available in the public database (http://www.ocri-genomics.org/bolbase/; http://brassicadb.org/brad/; and http://planttfdb.cbi.edu.cn/, respectively). The micro array data set supporting the result of expression data were downloaded from the AtGenExpress visualization tool (AVT) (http://jsp.weigelworld.org/expviz/expviz.jsp).

## Competing interests

The authors declare that they have no competing interests.

## Authors’ contributions

All the authors read and approved the final manuscript. The study was conceived by ISN and JIP. SKTA collected the public dataset of researched and data analysis, bioinformatics analysis, and manuscript preparation. ISN and JIP participated in cabbage and other species planning of analysis and revising the manuscript. HJJ participated in samples collection, data analysis and qPCR validation. ISN and JIP participated in data analyses and revised the manuscript.

## Supplementary Material

Additional file 1: Table S1 Complete list of ERF/AP2 transcription factors identified in the cabbage genome from bolbase (http://www.ocri-genomics.org/bolbase/). **Table S2.** Best BLAST hits of AP2/ERF subfamily domain genes of Chinese cabbage and *Arabidopsis* used for phylogenetic tree construction. **Table S3.** Chromosome wise distribution of superfamily genes. **Table S4.** The paralogous genes of the AP2/ERF superfamily in cabbage. **Table S5.***B. oleracea* AP2/ERF superfamily gene expression profiles in abiotic stresses. The expression data were downloaded from the AtGenExpress visualization tool (AVT) (http://jsp.weigelworld.org/expviz/expviz.jsp). **Table S6.** The annotations of all the AP2/ERF genes of cabbage in non-redundant databases from blast2go (http://www.blast2go.com). **Table S7.** List of primers used for RT-PCR and real-time PCR analysis. Click here for file

Additional file 2: Figure S1 Classification of AP2/ERF family transcription factors in cabbage. The size of each segment is proportional to the relative abundance of the assigned AP2/ERF factor. **Figure S2.** Phylogenetic tree constructed by the neighbor-joining method using AP2 family transcription factor domains in cabbage, Chinese cabbage and *Arabidopsis*. The numbers are bootstrap values based on 1000 iterations. **Figure S3.** The DREB subfamily protein motifs derived from each species. Overall the stack indicates the sequence conservation. The height of residues within the stack indicates the relative frequency of each residue at the position. **Figure S4.** The ERF subfamily protein motifs derived from each species examined. **Figure S5.** The RAV, AP2 and Soloist family protein motifs derived from each species examined. **Figure S6.** AP2/ERF protein motifs from each of the species examined. **Figure S7.** Expression profile cluster analyses of cabbage ERF subfamily genes. **Figure S8.** Expression profile cluster analyses of cabbage RAV family genes. **Figure S9.** Expression profile cluster analyses of cabbage AP2 family genes. **Figure S10 (a-e).** Identification and characterization of *BoCBF* genes in *B. oleracea*, results were revealed based on previous studies [48,49]. Detailed description of each figures were described in the ppt section. Click here for file

Additional file 3 Cold, Salt, drought and ABA. Click here for file
