# Supplementary material for: Genome-wide analysis of the distribution of AP2/ERF transcription factors reveals duplication and CBFs genes elucidate their potential function in Brassica oleracea
Source: BMC Genomics. 2014 Jun 3;15:422. doi: 10.1186/1471-2164-15-422 (PMC4229850; doi:10.1186/1471-2164-15-422)
Supplement: Additional file 2: Figure S1 — Classification of AP2/ERF family transcription factors in cabbage. The size of each segment is proportional to the relative abundance of the assigned AP2/ERF factor. Figure S2. Phylogenetic tree constructed by the neighbor-joining method using AP2 family transcription factor domains in cabbage, Chinese cabbage and Arabidopsis. The numbers are bootstrap values based on 1000 iterations. Figure S3. The DREB subfamily protein motifs derived from each species. Overall the stack indicates the sequence conservation. The height of residues within the stack indicates the relative frequency of each residue at the position. Figure S4. The ERF subfamily protein motifs derived from each species examined. Figure S5. The RAV, AP2 and Soloist family protein motifs derived from each species examined. Figure S6. AP2/ERF protein motifs from each of the species examined. Figure S7. Expression profile cluster analyses of cabbage ERF subfamily genes. Figure S8. Expression profile cluster analyses of cabbage RAV family genes. Figure S9. Expression profile cluster analyses of cabbage AP2 family genes. Figure S10 (a-e). Identification and characterization of BoCBF genes in B. oleracea, results were revealed based on previous studies [48,49]. Detailed description of each figures were described in the ppt section. [file 1471-2164-15-422-S2.pptx]

## Slide 1
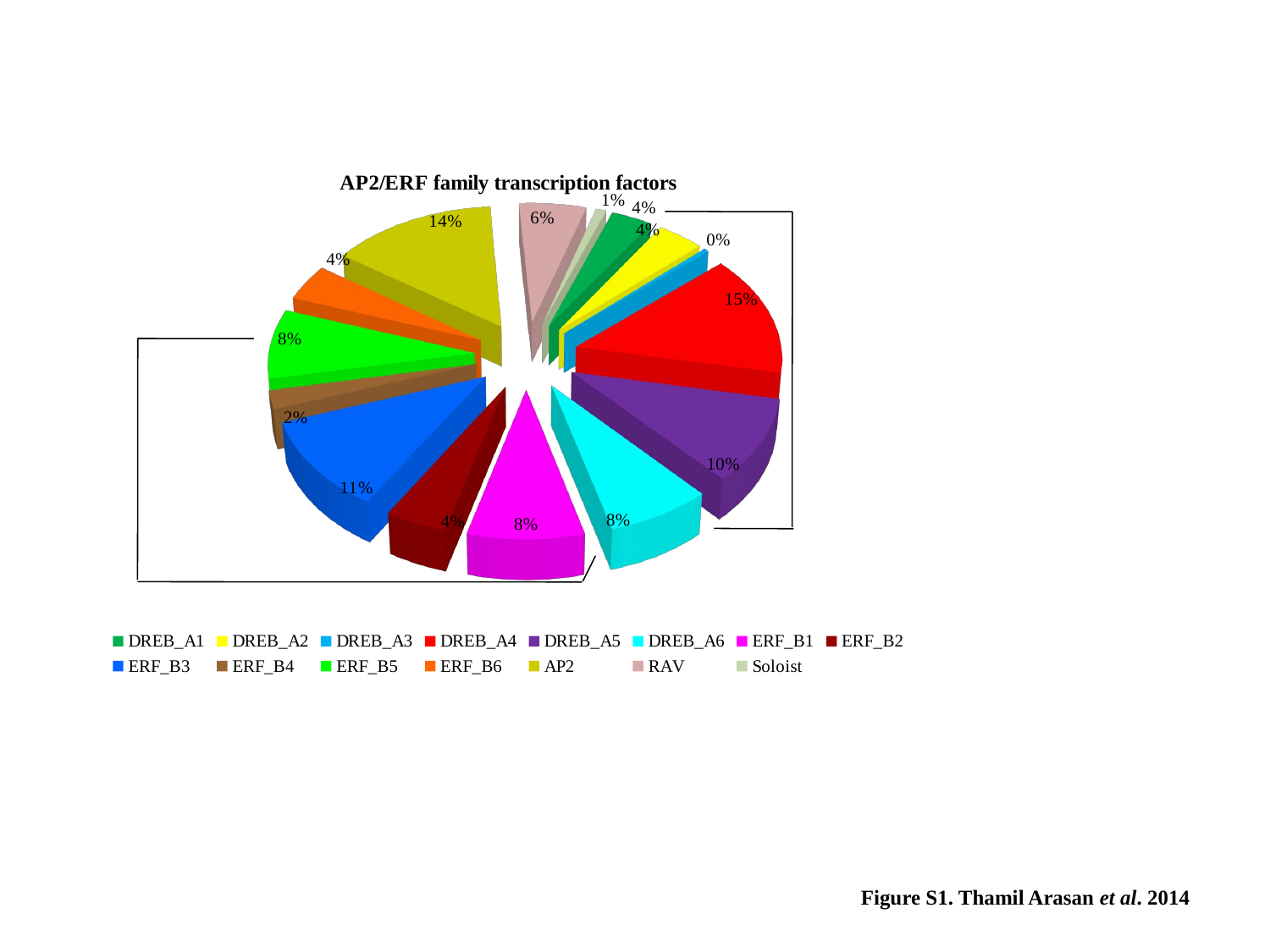

[unsupported chart]
Figure S1. Thamil Arasan et al. 2014

## Slide 2
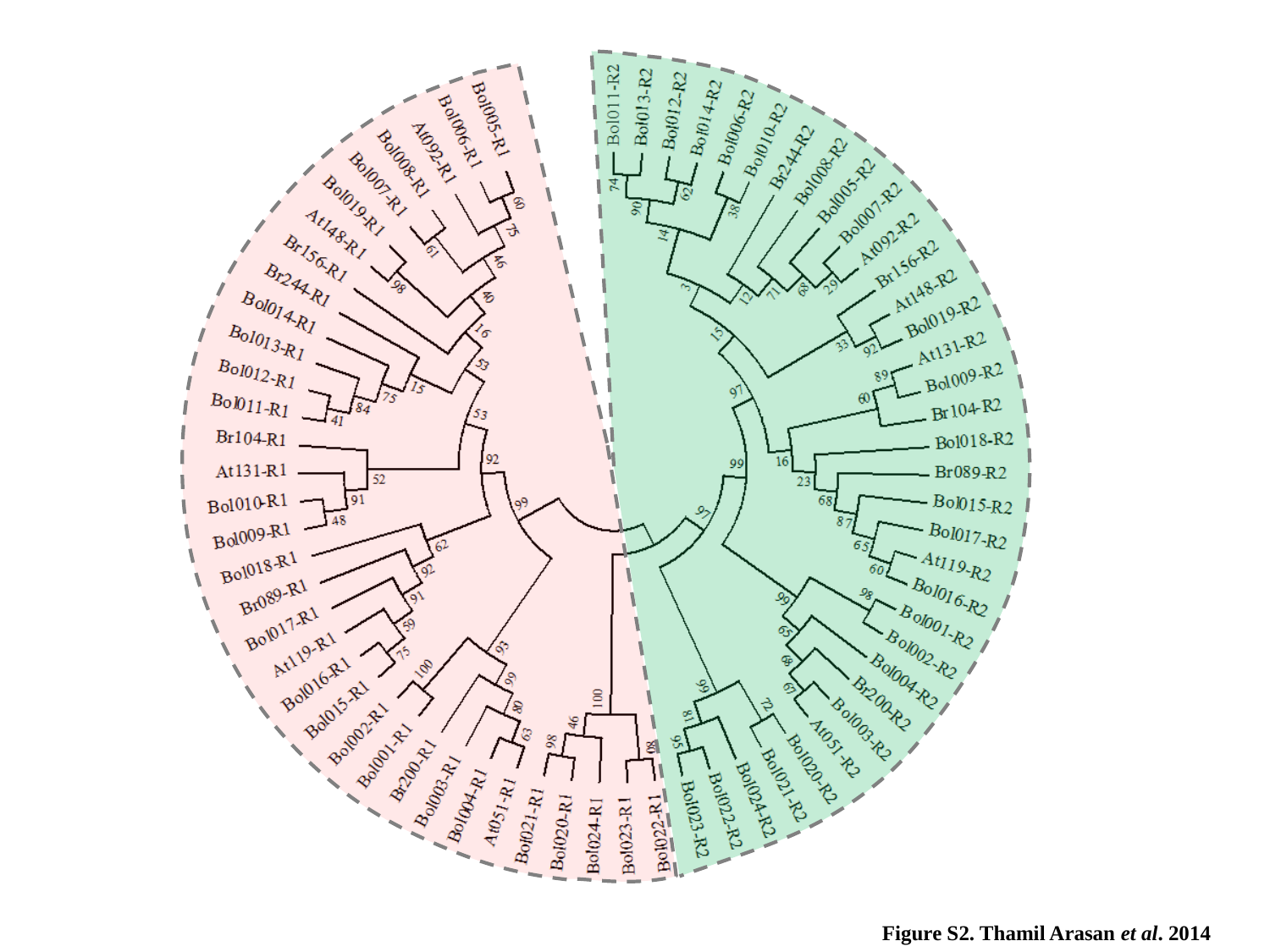

Figure S2. Thamil Arasan et al. 2014

## Slide 3
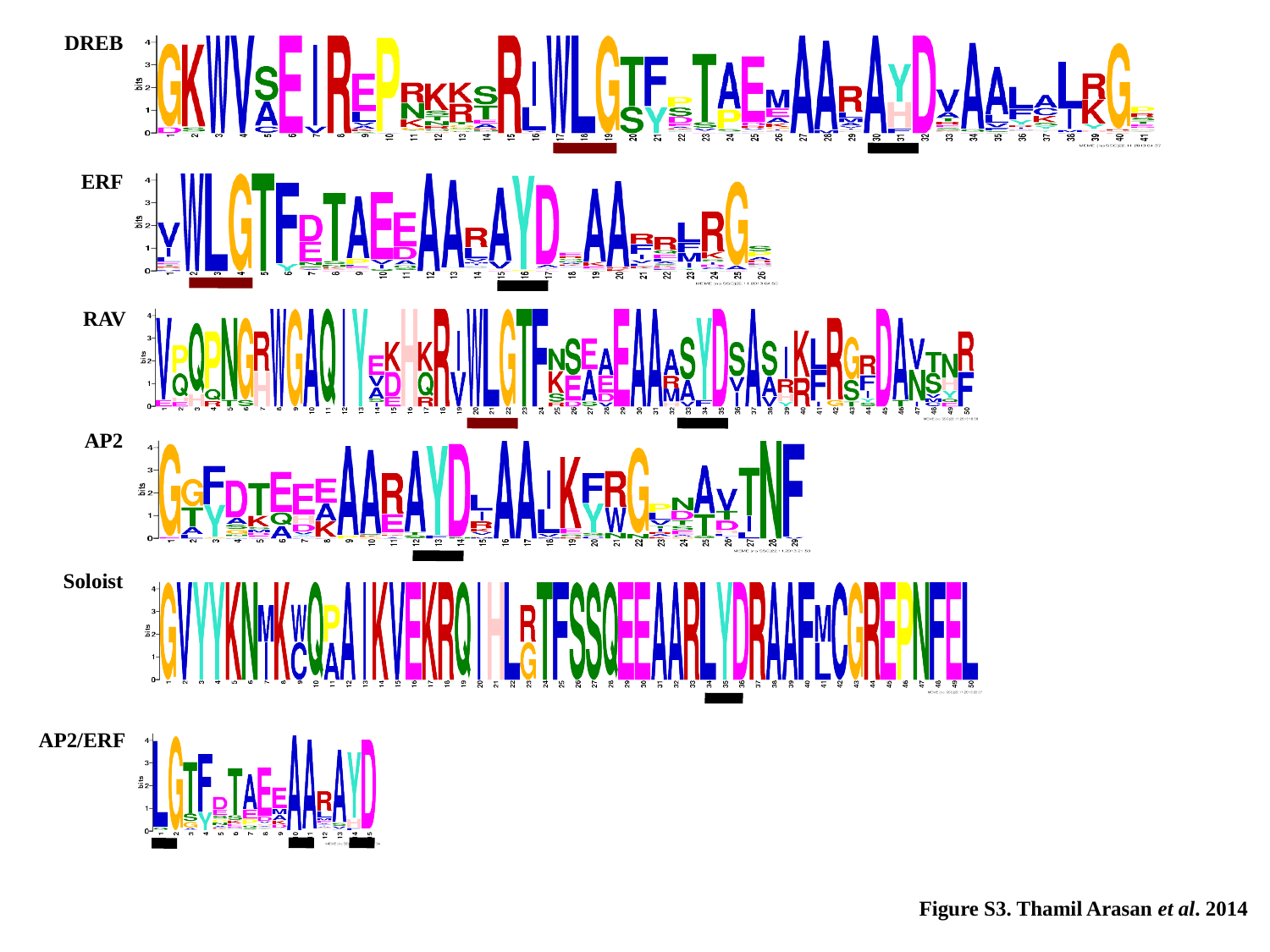

DREB
ERF
RAV
AP2
Soloist
AP2/ERF
Figure S3. Thamil Arasan et al. 2014

## Slide 4
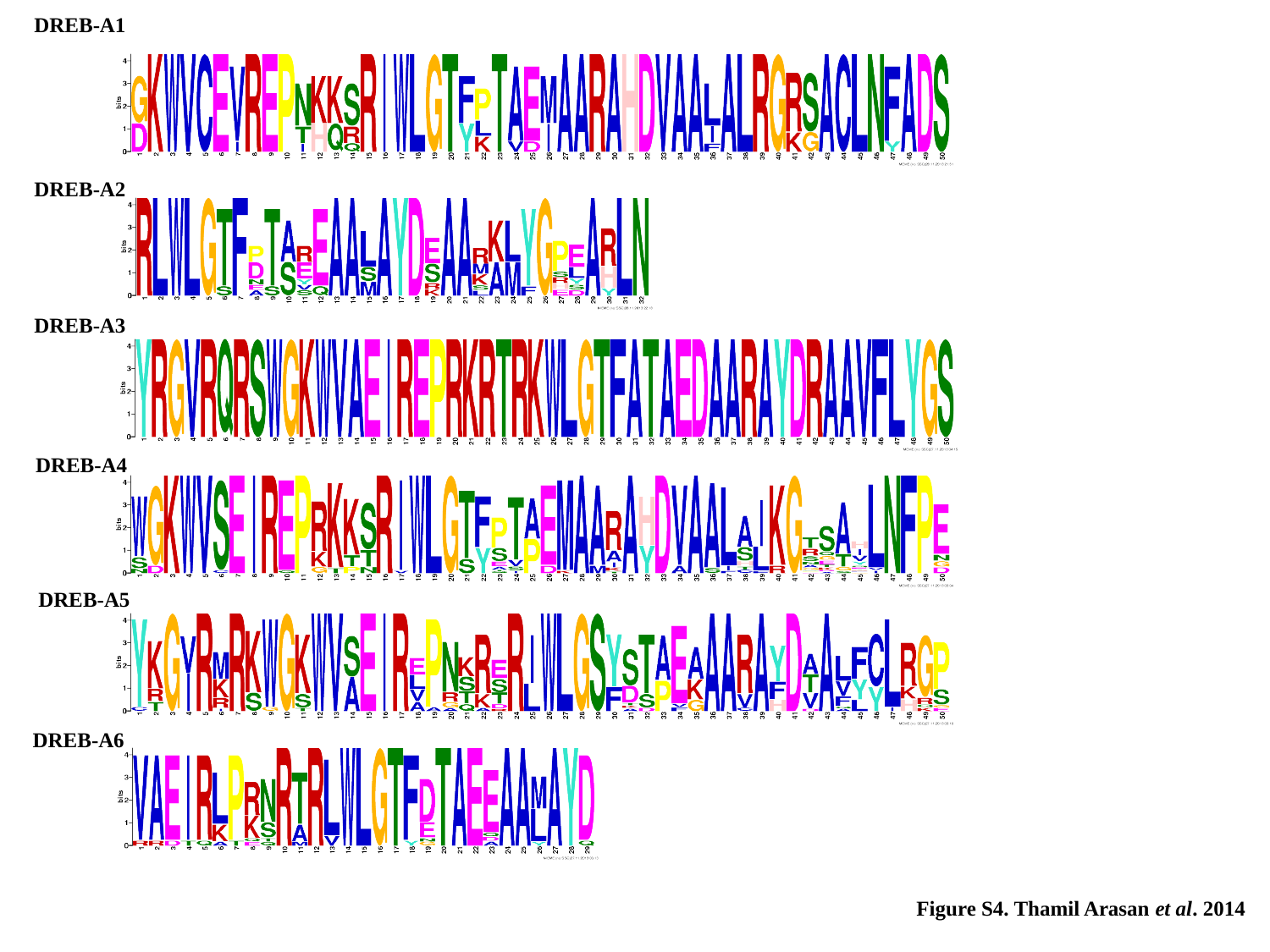

DREB-A1
DREB-A2
DREB-A3
DREB-A4
DREB-A5
DREB-A6
Figure S4. Thamil Arasan et al. 2014

## Slide 5
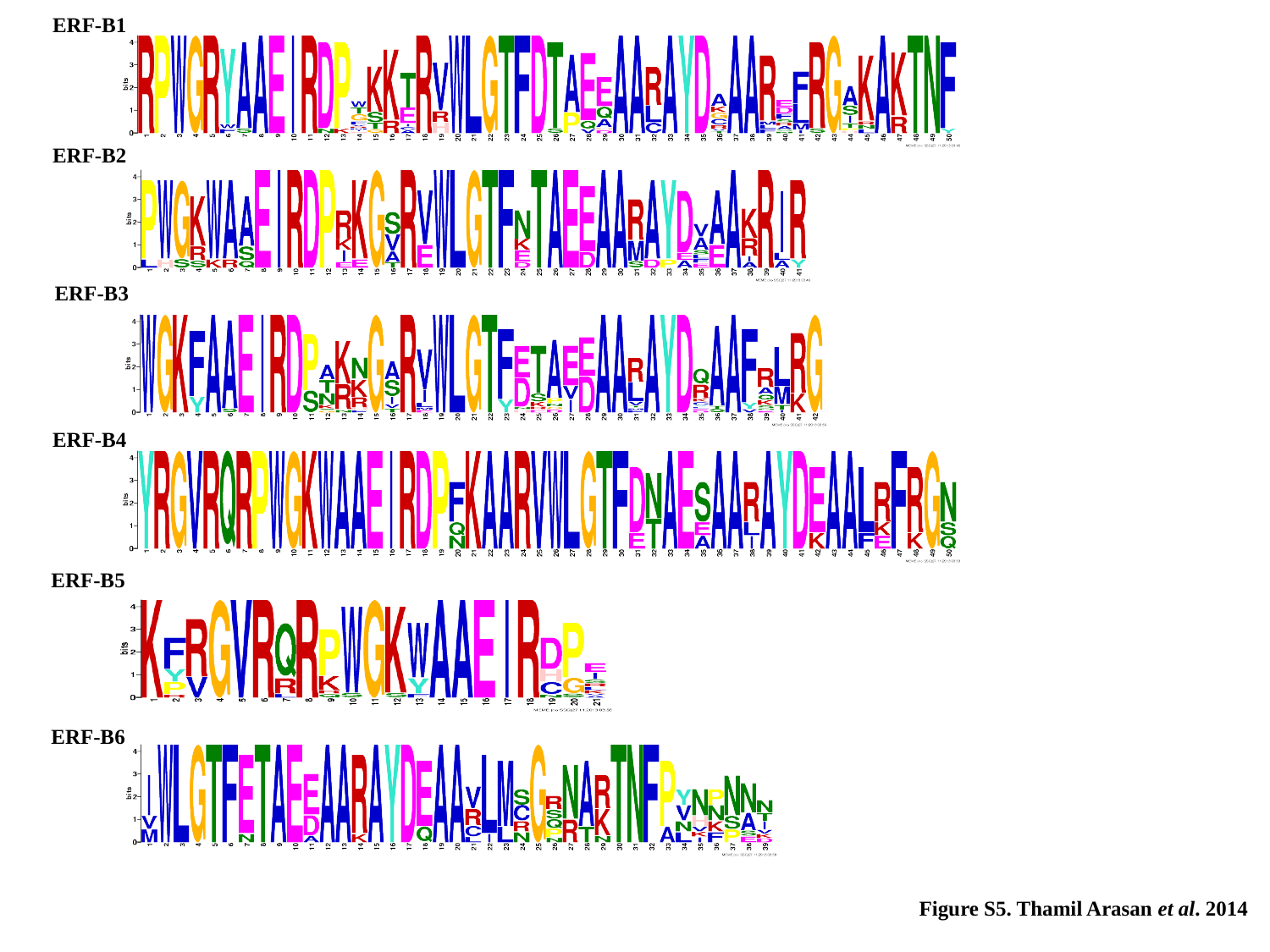

ERF-B1
ERF-B2
ERF-B3
ERF-B4
ERF-B5
ERF-B6
Figure S5. Thamil Arasan et al. 2014

## Slide 6
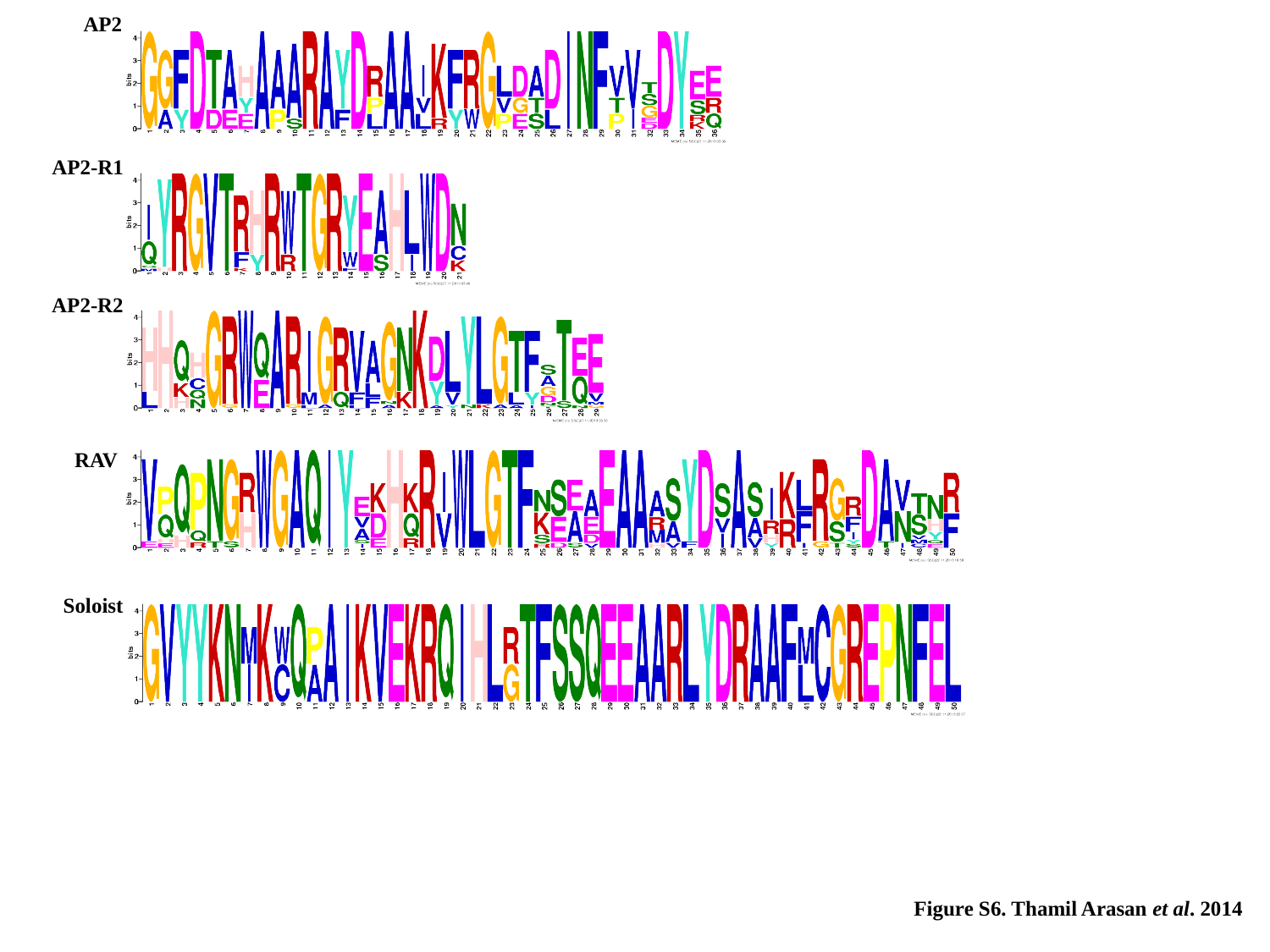

AP2
AP2-R1
AP2-R2
RAV
Soloist
Figure S6. Thamil Arasan et al. 2014

## Slide 7
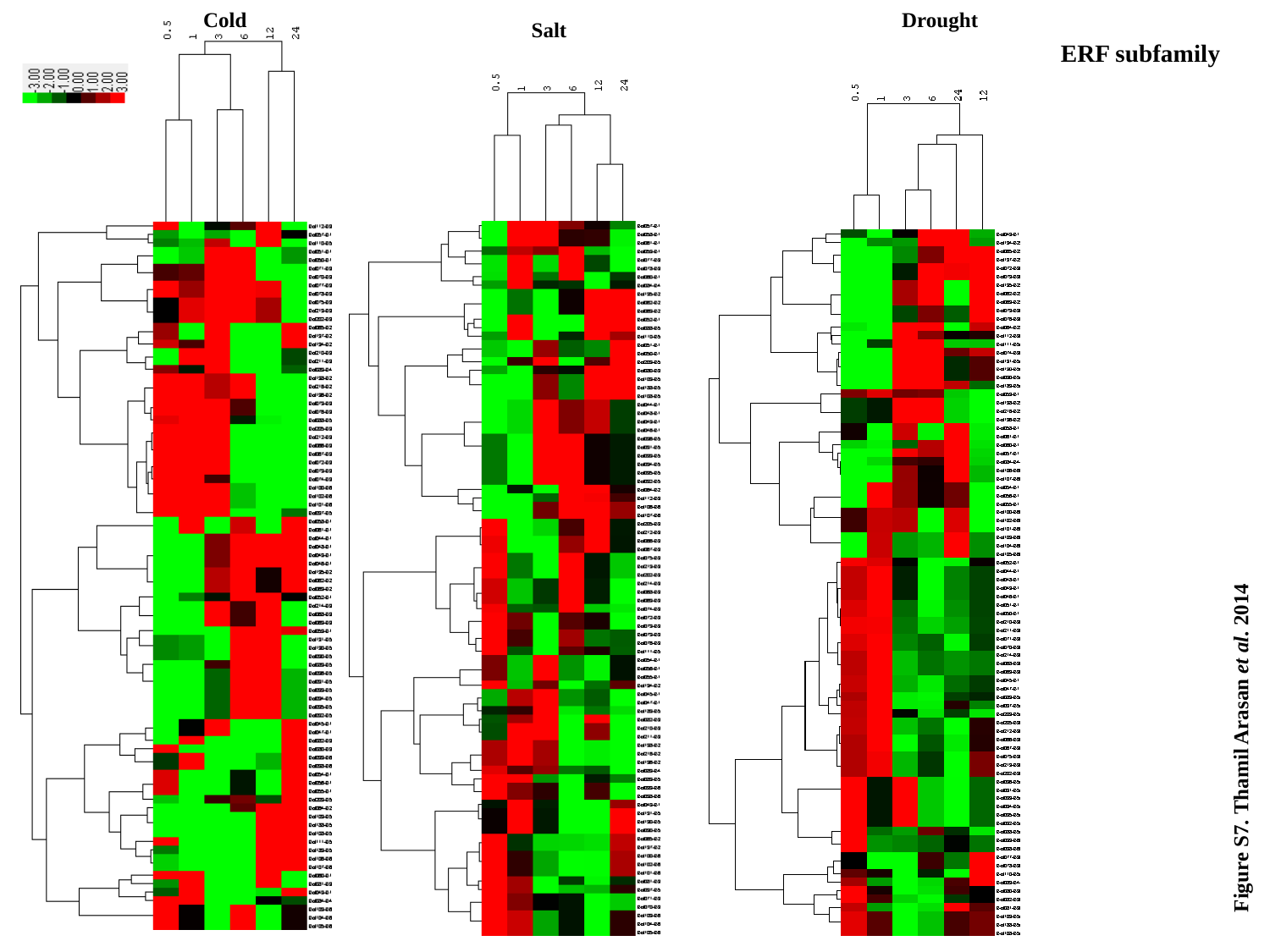

Cold
Drought
Salt
ERF subfamily
Figure S7. Thamil Arasan et al. 2014

## Slide 8
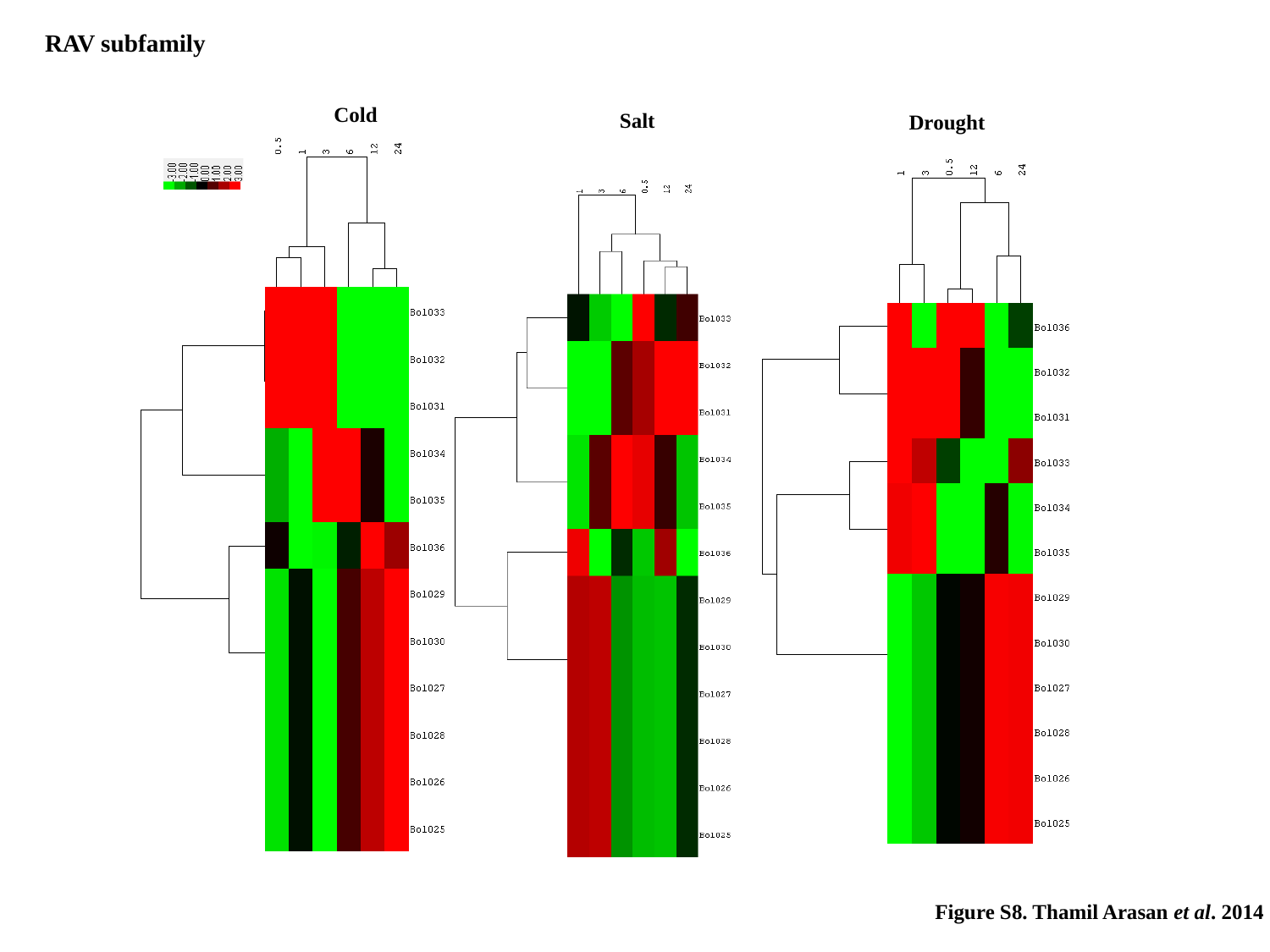

RAV subfamily
Cold
Salt
Drought
Figure S8. Thamil Arasan et al. 2014

## Slide 9
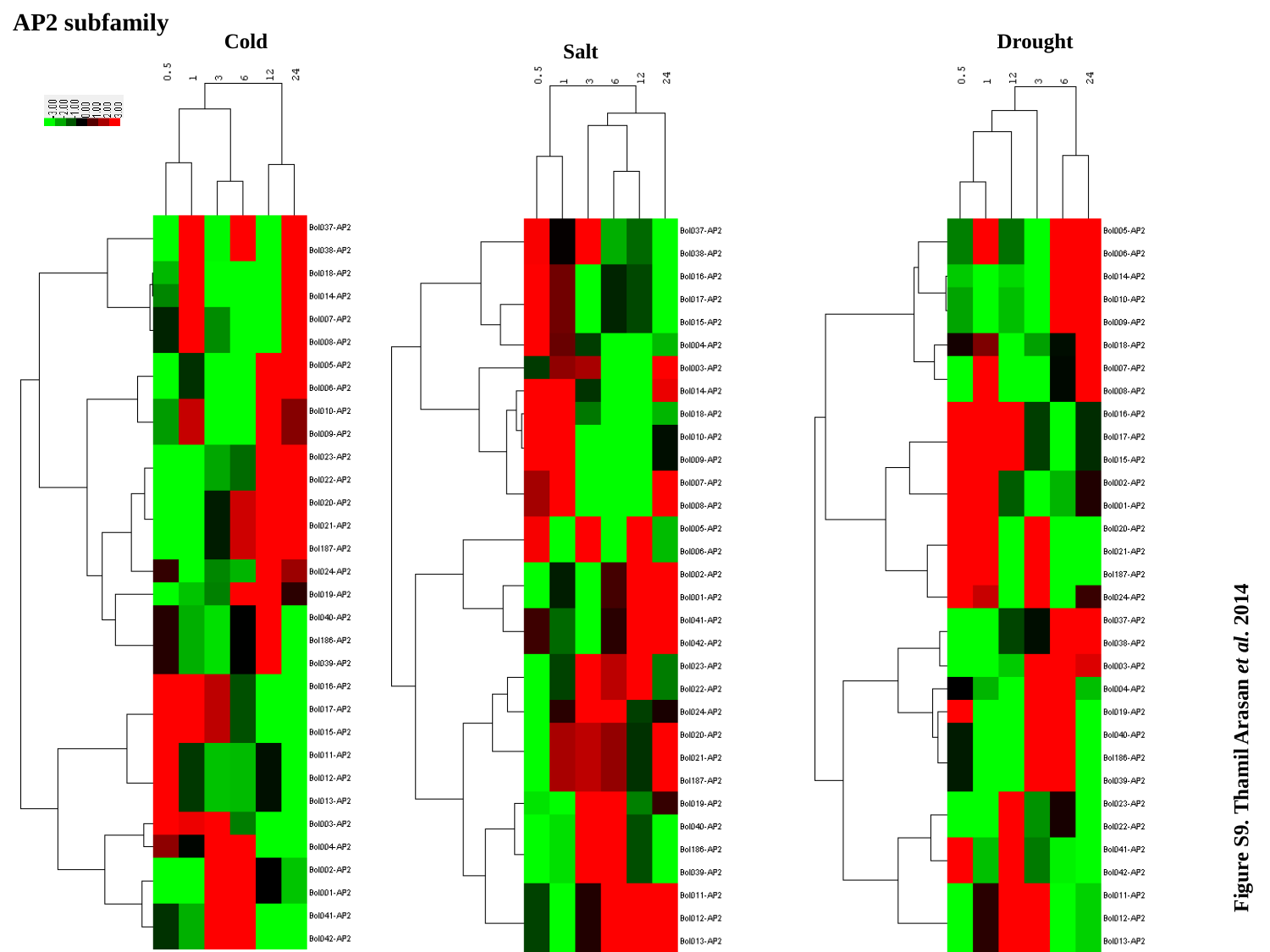

AP2 subfamily
Cold
Drought
Salt
Figure S9. Thamil Arasan et al. 2014

## Slide 10
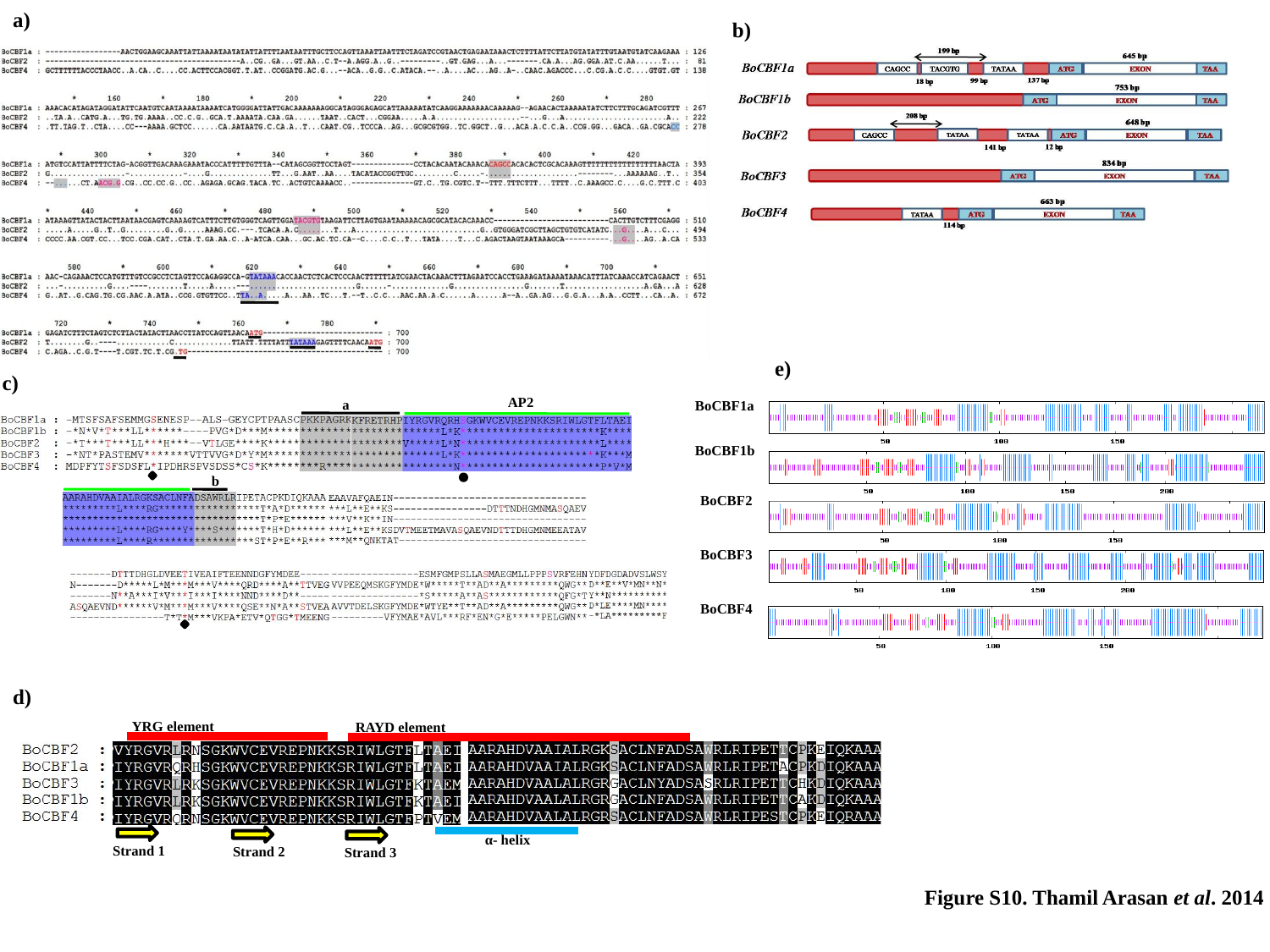

a)
b)
e)
c)
AP2
a
b
BoCBF1a
BoCBF1b
BoCBF2
BoCBF3
BoCBF4
d)
YRG element
RAYD element
α- helix
Strand 1
Strand 2
Strand 3
Figure S10. Thamil Arasan et al. 2014
